# Dietary Lipid:Protein Ratio and n-3 Long-Chain Polyunsaturated Fatty Acids Alters the Gut Microbiome of Atlantic Salmon Under Hypoxic and Normoxic Conditions

**DOI:** 10.3389/fmicb.2020.589898

**Published:** 2020-12-23

**Authors:** David Huyben, Beeke K. Roehe, Michaël Bekaert, Bente Ruyter, Brett Glencross

**Affiliations:** ^1^Institute of Aquaculture, University of Stirling, Stirling, United Kingdom; ^2^Department of Animal Biosciences, University of Guelph, Guelph, ON, Canada; ^3^Norwegian Institute of Food, Fisheries, and Aquaculture Research (Nofima), Tromsø, Norway

**Keywords:** bacteria, hypoxia, intestinal microbiota, salmonids, predictive metagenomic function, 16S rRNA gene

## Abstract

Researchers have adjusted dietary lipid:protein ratios and n-3 long-chain polyunsaturated fatty acids (LC-PUFA) to optimize the growth performance of Atlantic salmon. However, dietary impacts on the gut microbiome are lacking, especially under varying environmental conditions. To examine this response, post-smolt salmon (184 ± 5 g) were fed diets with lipid:protein ratios considered low (180, 570 g/kg) and high (230, 460 g/kg) along with low and high levels of n-3 LC-PUFA (7 or 14 g/kg) while fish were reared under low and high levels of dissolved oxygen (6.7 or 8.0 mg/L). At day 0, 35 and 116, digesta in the distal intestine were collected and analyzed for viable counts and 16S ribosomal RNA (rRNA) genes (V4 region) using Illumina MiSeq. The reduction in oxygen had negligible effects, except on viable plate counts of total bacteria and an initial effect on beta-diversity. In contrast, the high lipid (HL) diets had an increased alpha-diversity (e.g., Shannon and Chao-1) at day 0 and day 35 whereas high n-3 diets suppressed these indices at day 116. Generally, a reduction in alpha-diversity was observed over time and an interaction between lipid:protein ratio x n-3 was found. Between diets, beta-diversity and phyla abundance were similar as both Proteobacteria (44%) and Firmicutes (21%) dominated. However, at the genus level *Aliivibrio*, *Streptococcus*, Weissella, and *Lactobacillus*, were associated with low lipid (LL) diets while the high lipid diets were associated with less abundant bacteria, e.g., *Chromohalobacter*. At day 116, the relative abundance of the Tenericutes phylum increased 10-fold (36%). Fish fed the high lipid diet with high n-3 had reduced alpha-diversity, lowest abundance of lactic acid bacteria, and highest abundance of *Mycoplasma*, which may indicate a less healthy gut microbiome. Phylogenetic Investigation of Communities by Reconstruction of Unobserved States (PICRUSt) analysis revealed that saturated and unsaturated fatty acid biosynthesis pathways were several folds higher in fish fed the high lipid diet, possibly to compensate for the lack of dietary n-3. In summary, our results show that the viable plate counts, alpha-diversity, beta-diversity, and predictive function of gut bacteria in Atlantic salmon post-smolts are influenced by dietary lipid:protein ratio and n-3 LC-PUFA over several time points with little effect by dissolved oxygen.

## Highlights

- Dissolved oxygen level had little to no effect on the gut microbiome.
- Alpha-diversity was affected initially by lipid:protein ratio, but later by n-3 LC-PUFA level.
- High lipid diets had reduced abundance of lactic acid bacteria replaced by *Mycoplasma*.
- Fish fed low n-3 had more gut bacteria related to fatty acid biosynthesis pathways.

## Introduction

The gut microbiota (e.g., bacteria and fungi) play a vital role in fermenting dietary carbohydrates and producing short chain fatty acids (SCFA), such as acetate, propionate, and butyrate, which can be utilized in energy metabolism and enhance gut health (Kihara and Sakata, 1997; Asaduzzaman et al., 2018). Abundance of different phyla in the gut (e.g., Firmicutes) has been found to increase lipid deposition in zebrafish (*Danio rerio*; Semova et al., 2012). It is important to better understand the interactions between diet, gut microbes and growth performance of Atlantic salmon (*Salmo salar*) because they are an economically important fish to the aquaculture industry. Only two studies have used next-generation sequencing to determine the influence of dietary lipids on the salmon gut microbiota (Zarkasi et al., 2016; Rudi et al., 2018). Feeding high lipid:protein diets was found to increase the diversity and abundance of lactic acid bacteria in the gut of Atlantic salmon (Zarkasi et al., 2016). However, energy levels were not equal between diets and the gut bacteria were not associated with any functional role, e.g., using a predictive metagenomic approach.

Researchers have adjusted dietary levels of lipids, protein, and n-3 long-chain polyunsaturated fatty acids (LC-PUFA), such as eicosapentaenoic acid (EPA) and docosahexaenoic acid (DHA), to optimize the growth performance of Atlantic salmon (*Salmo salar*; Ruyter et al., 2000; Glencross et al., 2014; Bou et al., 2017). However, only a few studies have investigated the effects of lipid and n-3 LC-PUFA on the gut microbiome of salmon, which mainly look at replacing fish oil with vegetable oils as a sustainable alternative (Ringø et al., 2018). Feeding oils with different levels of n-3 and n-6 PUFA were found to influence viable counts of both lactic acid and pathogenic bacteria in the gut of Arctic charr (*Salvelinus alpinus*; Ringø, 1993; Ringø et al., 2002). However, replacing fish oil with vegetable oils was found to have minor changes on the gut microbiota of rainbow trout fry (*Oncorhynchus mykiss*; Ingerslev et al., 2014) and Atlantic salmon pre‐ and post-smolts (Rudi et al., 2018) using 16S rRNA gene next-generation sequencing. However, effects on gut microbiota may have been masked due to short experiment duration or fish life-stage changes. In addition, reductions in dissolved oxygen (i.e. hypoxia) have been found to change gut microbiota in temperate fish and shrimp (Fan et al., 2020; Sun et al., 2020). Hypoxia is a common stressor in salmon farm operations known to reduce feed intake (Huyben et al., 2021), although research on the hypoxic effects on gut microbial populations is lacking.

The objective of this study was to characterize the gut microbiota of Atlantic salmon at three different time points and determine if the viable plate counts, alpha-diversity, beta-diversity, and predictive function of gut microbes can be influenced by the dietary lipid:protein ratio, level of n-3 PUFA, and dissolved oxygen. We hypothesized that high levels of n-3 PUFA and hypoxia influence the growth of certain gut microbes and reduce the alpha-diversity based on reduced reliance on fatty acid producing bacteria and reduced feed intake.

## Materials and Methods

### Fish and Facilities

In May 2018, Atlantic salmon post-smolts were acquired from a local producer and reared at the Marine Environmental Research Laboratory (Machrihanish, United Kingdom). Fish were randomly allocated to 24 circular tanks containing 40 fish each. Each 500 L tank was equipped with a LED light, air stone, and automated feeder (Arvo-tec Oy, Huutokoski, Finland). Fish (184.4 ± 4.9 g; mean ± SD) were acclimated on a commercial diet (3 mm Intro, Biomar Ltd., Grangemouth, United Kingdom) for 3 weeks before the start of the experiment. During the 116 day (17 week) experiment, fish were fed twice per day for 3 h durations and the uneaten feed waste was collected each morning to allow for estimation of daily feed intakes in each tank. The flow rate and aeration were reduced in half of the tanks to apply a hypoxic stressor. The low oxygen tanks had a dissolved oxygen concentration of 78.0 ± 2.3% (6.7 ± 0.2 mg/L) compared to an oxygen saturation of 92.6 ± 2.7% (8.0 ± 0.2 mg/L) for the high oxygen tanks. The filtered seawater had a temperature of 13.2 ± 0.2°C (mean ± SD). This experiment was approved by the Animal Welfare and Ethical Review Body (reference AWERB/1617/84) in accordance with the United Kingdom Home Office under the Animals (Scientific Procedures) Act 1986.

Groups of fish were fed one of four diets that were formulated to have markedly different lipid:protein ratios (26.2 g/MJ or 21.0 g/MJ) based on combinations of high protein (570 g/kg) and low lipid (LL; 180 g/kg), or low protein (460 g/kg) and high lipid (HL; 230 g/kg), but were all formulated to be isoenergetic on a digestible basis (21 MJ DE/kg). Additionally, each set of diets had secondary treatments that varied in their level of n-3 PUFA (7 or 14 g/kg). The 3 mm pellets were produced using extrusion processing by a commercial manufacturer (Sparos I&D, Olhão, Portugal).

### Sample Collection

Fish at day 21 (fed commercial diet), 0 (initial feeding of test diets), 35 (short-term feeding), and 116 (long-term feeding) were overdosed with tricaine methanesulfonate (MS-222), cervical vertebrae were dislocated, and their abdomen was dissected. A sterile scalpel blade was used to cut the distal intestine at the ileo-rectal valve and 0.5 cm before the anus. Using sterile forceps, the fecal content (hindgut digesta) was firmly squeezed into a sterile cryotube in order to collect both autochthonous and allochthonous bacteria, frozen on dry ice, and stored at −70°C. Nine fish were randomly sampled at day 21 (*N* = 9), three from each diet at day 0 (*N* = 27), and nine from each diet at day 35 (*N* = 72) and 116 (*N* = 72). In addition, triplicate samples of each experimental diet and the commercial diet were collected from newly opened bags, homogenized, frozen, and stored at −70°C. At day 116, the tanks were drained and a sterile cotton swab was used to collect biofilm from the side of three randomly chosen tanks to act as an environmental sample, which was frozen and stored at −70°C.

### Viable Plate Counts of Bacteria

Plate counts of viable bacteria were performed on two different media of either typtone soya agar (TSA; Sigma-Aldrich, Dorset, United Kingdom) or Man-Rogosa-Sharpe agar (MRS; Sigma-Aldrich) to isolate total and lactic acid bacteria on days 21, 0, 35, and 116. First, approximately 100 mg of feces was added to 900 μl sterile 0.85% NaCl, vortexed, and serial diluted. Four droplets of 10 μl each were pipetted onto agar plates for each dilution, dried at room temperature for 2 h, sealed with parafilm, and then incubated at 22°C for 4–6 days. Mean counts per droplet were divided by the dilution factor of both the droplet volume and dilution series to calculate total viable counts as colony forming units (CFUs) per ml^−1^. Some viable plate count samples were removed due to contamination or overgrowth of bacteria and fungi.

### 16S rRNA Gene Extraction, Amplification, and Sequencing of Bacteria

Processing and sequencing of bacteria were performed on two separate occasions; (1) day 0 and 35 gut samples and (2) day 116 gut and environmental samples. Day 21 samples were not included since their composition would be similar to day 0. Approximately 150 mg of feces, diet, and tank biofilm swab were each homogenized in 1 ml of Inhibit EX buffer (Qiagen Ltd., Manchester, United Kingdom) using a Mini-bead-breaker 16 (Biospec Products Inc., Bartlesville, OK, United States) for four cycles of 30 s with 60 s rest. The DNA was extracted using QIAamp Fast DNA Stool Mini kit (Qiagen Ltd.) according to the manufacturer’s instructions. In addition, samples were incubated for 10 min at 95°C to reduce foam formation after homogenization and to enhance lysis of Gram-positive bacteria. Two samples of nuclease free water were extracted, amplified, and sequenced to represent negative controls and to identify potential contaminants. Samples were eluted with 20 μl of nuclease free water into new tubes. The nucleic acid and DNA concentrations were quantified using a ND-1000 Nanodrop spectrophotometer (Nanodrop Technologies LLC, Wilmington, DE, United States) and a Quibit 2.0 fluorimeter (Invitrogen, Thermo Fisher Scientific, Hempstead, United Kingdom). Samples were diluted to a concentration of 25 ng/ml of nucleic acid (Nanodrop) or 2.5 ng/ml of DNA (Quibit).

A PCR was performed on 25 μl reactions consisting of 2 μl template (5 ng of DNA), 1.25 μl (10 μM) of each forward primer (515F; GTGYCAGCMGCCGCGGTAA) and reverse primer (806R; GGACTACNVGGGTWTCTAAT; Caporaso et al., 2011) with Illumina adapter to target the V4 region of the 16S rRNA gene, nuclease free water, and 12.5 μl of 2x NEBNext Ultra II Q5 Master Mix (New England Biolabs Ltd., Hitchin, United Kingdom). The PCR was performed in a T-advanced thermocycler (Biometra GmbH, Göttingen, Germany) under condition: 98°C for 60 s followed by 30 cycles of 98°C for 10 s, 53°C for 10 s, and 65°C for 45 s with a final step of 65°C for 5 min. Amplicons were confirmed on a 1% agarose gel alongside negative controls of nuclease free water instead of template. Samples were purified with Axygen AxyPrep Mag PCR clean up kit (Corning Inc., Corning, NY, United States) using magnetic beads at a ratio of 1:1 and two washes of 70% EtOH, according to the manufacturer’s instructions. Samples were eluted with 20 μl of 10 mM Tris (Qiagen Ltd.) and 7.5 μl was used in a second PCR with the above conditions, except only for 10 cycles and the forward and reverse primers consisted of different combinations of eight basepairs from the Nextera XT DNA Library Preparation kit (Illumina Inc., Cambridge, United Kingdom) to individually index each sample. Samples were purified with Axygen magnetic beads (Corning Inc.) as above, quantified with a Quibit 2.0 fluorimeter (Thermo Fisher Scientific), and diluted with 10 mM Tris (Qiagen Ltd.) to a concentration of 2.81 ng/μl or 10 nM based on a final PCR product of 428 basepairs and a molar weight of 656.6 M. An equal volume of each sample was pooled, confirmed on a 1% agarose gel, and sequenced on the Illumina MiSeq platform at the University of Stirling (Stirling, United Kingdom) with a MiSeq Reagent kit v2 of 500 cycles (Illumina Inc.).

### Bioinformatics

The 16S rRNA gene sequences were analyzed using Mothur version 1.42.3 (Schloss et al., 2009) according to the MiSeq SOP (https://www.mothur.org/wiki/MiSeq_SOP; Kozich et al., 2013). Sequence reads which were smaller than 200 bp, larger than 300 bp, had more than eight consecutive bp and were outside the V4 region of the 16S rRNA gene were removed from the dataset. Filtered sequence reads were aligned to the SILVA reference database version 123 (Quast et al., 2012), pre-clustered to merge sequences with less than 2 bp difference, and chimeras were removed using the open-source tool VSEARCH (Rognes et al., 2016). Sequences were classified using the RDP Bayesian Classifier trainset version 16.0 at a cut off of 80% (Cole et al., 2013), and taxon resembling chloroplasts, mitochondria, unknowns, archaea, and eukaryotes were removed. Count tables of unique sequences were created to reduce processing time. Sequences were clustered and classified into operational taxonomic units (OTUs) at a cut off of 0.03. *Ralstonia* (genus level) was identified as a contaminant based on previous research on common laboratory contaminants in microbiome studies (Salter et al., 2014) and its high abundance of 84 and 53% on days 0/35 and 116 in the blank samples, thus *Ralstonia* was removed from both datasets. Sequences were subsampled in order to normalize all samples to the lowest number of sequences per samples, which was 8,257 and 8,072 for day 0/35 and 116 datasets, respectively. The raw 16S rRNA gene sequence reads were deposited in the Sequence Read Archive of NCBI and made publicly available under BioProject Accession number PRJNA650141.^1^

### Statistical Analysis

Normal distribution and homogeneity of each dataset were determined using Shapiro-Wilk and Levene tests in R version (R-Core-Team, 2015). When needed, data were normalized by log, square-root, or arcsine transformation. All data are presented as means ± SE unless otherwise specified. Akaike Information Criterion (AIC) was used to determine the statistical model that best fit the data. Differences between treatments were determined using linear mixed effects (*lme*) or linear model (*lm*) based on the *nlme* and *stats* packages in R v3.5 (Pinheiro et al., 2014). In *lme* model, fixed effects were lipids (lipid:protein ratio), n-3 PUFA and oxygen with fish tank as a random effect and interactions between all three factors (*y* = lipids × n3 × oxygen + 1 ¦ tank). In *lm* model, fixed effects and interactions were the same (*y* = lipids × n3 × oxygen). *p*-values from the models were generated using ANOVA tables and differences between treatments were determined using *lsmeans*. *p*-values below 0.05 were considered significant and *p*-values between 0.05 and 0.10 were considered to be a tendency.

For alpha-diversity, rarefaction.single, and summary.single commands in Mothur were used on subsampled OTU datasets to generate rarefaction curves and diversity tables based on Good’s coverage, observed species, Shannon diversity (non-parametric), and Chao-1 richness indices. These indices were analyzed to measure the total species represented, abundance/evenness of species, and the richness of rare species in the gut community. Significant differences between treatments were determined using *lme* and *lm* models in R as mentioned above. For beta-diversity, Bray-Curtis distance matrixes were square-root transformed and significance of each factor (e.g., lipid, n-3 PUFA, and oxygen) was determined using Analysis of Similarity (ANOSIM) with the *adonis* function based on the vegan package in R (Oksanen et al., 2018). Beta diversity was plotted using 2D Non-metric Multidimensional Scaling (NMDS). Linear discriminant analysis (LDA) Effect Size (LefSe) was used to identify OTUs that explain differences between treatments using Kruskal-Wallis tests and a LDA threshold of 2.0 (Segata et al., 2011).

### Predictive Metagenomic Analysis

In Mothur, the SILVA aligned and chimera-removed fasta file from the previous section was classified using the Greengenes reference database version gg_13_8_99 (Desantis et al., 2006), and non-bacteria were removed and the distance matrix was clustered as above. In order to use Phylogenetic Investigation of Communities by Reconstruction of Unobserved States (PICRUSt), an OTU table was generated using make.biom with Greengenes taxonomy and PICRUSt OTU map. In VirtualBox (Oracle, Redwood Shores, CA, United States), Microbiome Helper version 0.4 was used to normalize the OTU copy number across samples, make functional predictions using Kyoto Encyclopedia of Genes and Genomes (KEGG) database (Kanehisa and Goto, 2000) and collapse KEGG Orthologs into KEGG pathway predictions according to the PICRUSt tutorial (Langille et al., 2013). PICRUSt predictions were visualized using Statistical Analysis of Taxonomic and Functional Profiles (STAMP) software version 2.1.3 (Parks et al., 2014) by comparing each pair of treatments within each factor (*N* = 72) using extended error bar plots at three KEGG pathway levels. Significant differences for predictive metagenomic pathways were determined using Welch’s two-sided *t*-test (unequal variance) and a Bonferroni correction was applied to *p*-values to account for multiple testing.

## Results

### Growth Performance of Atlantic Salmon

Final weight was significantly affected by lipid:protein ratio (*p* = 0.031), n-3 (*p* = 0.007), and oxygen (*p* < 0.001) while feed intake was only affected by oxygen (*p* = 0.001). Final weight was highest for fish fed the high lipid diet with high n-3 LC-PUFA under high oxygen (552 ± 4 g/fish, mean ± SE) with a feed intake of 318 ± 7 g/fish. The lowest final weight was for fish fed the low lipid diet with low n-3 under low oxygen at 436 ± 17 g/fish with a feed intake of 210 ± 16 g/fish. Further details on the phenomic responses of the trial are published by Huyben et al. (2021).

### Viable Plate Counts of Bacteria

**Figure 1:**
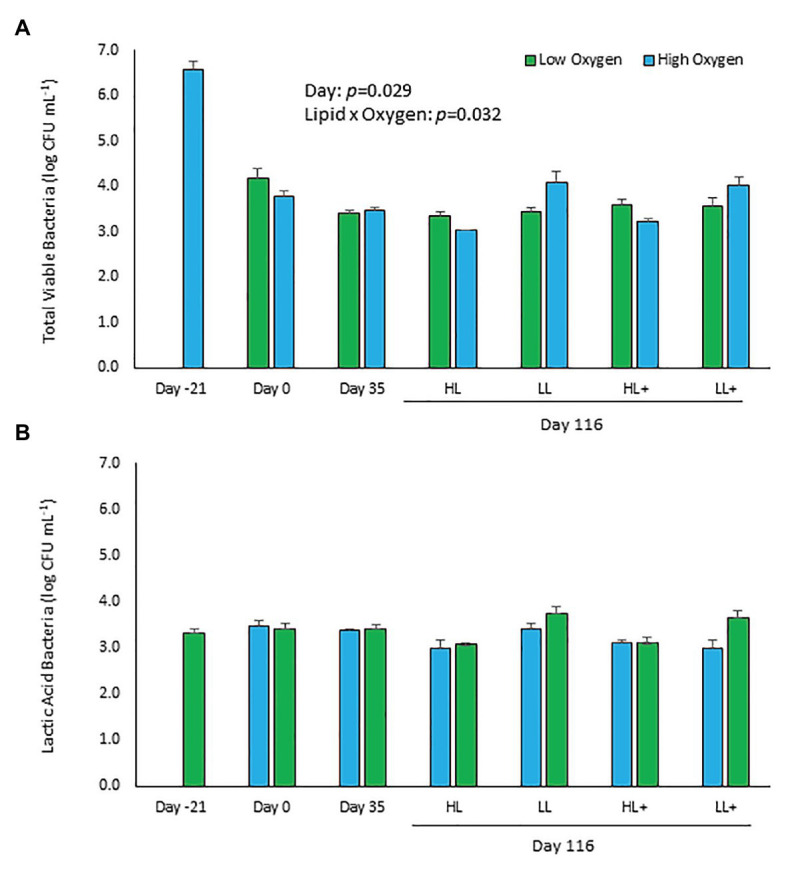
Viable plate counts of **(A)** total and **(B)** lactic acid bacteria (±SE) cultured on typtone soya agar (TSA) and man-rogosa-sharpe agar (MRS) plates of feces collected from the distal intestine of Atlantic salmon fed HL or LL diets with or without additional n-3 PUFA (+) and reared at low or high oxygen. Fish were sampled at -21 (*N* = 9), 0 (*N* = 23), 35 (*N* = 48), and 116 days (*N* = 55). Some samples excluded due to fungal overgrowth.

Total viable counts of aerobic bacteria cultured on TSA significantly decreased (*p* = 0.029) by approximately 3-logs over the course of the experiment from 3.7 ± 2.2 × 10^6^ (6.6 log) CFU ml^−1^ at day 21 compared to 4.2 ± 1.0 × 10^3^ (3.6 log) CFU ml^−1^ at day 116 (Figure 1A). No significant effects of lipid, n-3, or oxygen were found (*p* > 0.05), except for a significant lipid × oxygen interaction at day 116 (*p* = 0.032). Fish fed low lipid and kept at high oxygen had higher bacterial counts in their distal intestine.

Total viable counts of lactic acid bacteria cultured on MRS remained the same (*p* = 0.867) over the experiment with a mean count on day 116 of 2.9 ± 0.7 × 10^3^ (3.5 log) CFU ml^−1^ (Figure 1B). No significant effects of lipid, n-3, oxygen, or interactions between the three factors were found (*p* > 0.05).

### Alpha-Diversity of 16S rRNA Gene Sequences

**Figure 2:**
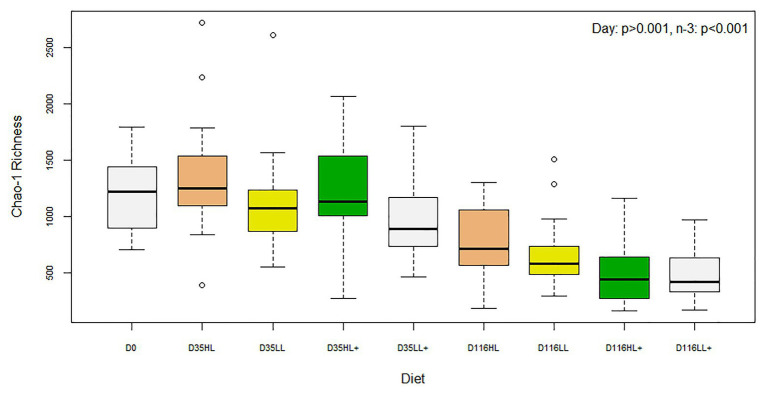
Box plots of bacterial alpha-diversity from the distal intestine of Atlantic salmon fed HL or LL diets with or without additional n-3 PUFA (+). Oxygen conditions were pooled together and fish were sampled on day 0 (*N* = 9), day 35 (*N* = 72), and day 116 (*N* = 72).

There was no difference in Shannon diversity or Chao-1 richness between fish gut bacteria sampled at day 0 and 35 (*p* = 0.155 and 0.682). On day 35, Good’s coverage, number of OTUs, Shannon diversity, and Chao-1 richness were all significantly affected by lipid level in the diet while no effects of n-3 or oxygen were found (Table 1). In general, alpha-diversity increased when fish were fed high lipid diets and the lowest diversity was found for fish fed low lipid diets at high oxygen. Fish fed the high lipid diet with low n-3 had the highest Shannon diversity at low oxygen and highest Chao-1 richness at high oxygen. All indices were similar between day 0 and 35 (*p* > 0.100), but both were significantly different (*p* < 0.05) compared to those on day 116 as a clear reduction in Chao-1 richness was found over time (Figure 2).

**Table 1:** Alpha-diversity indices of intestinal bacteria from Atlantic salmon fed high lipid (HL) or low lipid (LL) with or without additional n-3 polyunsaturated fatty acids [PUFA (+)] for 0 (*N* = 9) and 35 days (*N* = 72) and reared at low and high oxygen.

		Low oxygen				High oxygen				Pooled		*p*-value**		
	Day0^*^	HL	LL	HL+	LL+	HL	LL	HL+	LL+	SE	Lipid	n-3	Oxygen	X
Coverage (%)	96.5	96.6^ab^	96.2^ab^	96.4^ab^	97.0^ab^	95.7^a^	97.2^b^	96.5^ab^	97.3^b^	0.4	0.025	0.202	0.467	ns
No. of OTUs	478	525^ab^	541^b^	538^ab^	466^ab^	626^b^	412^a^	506^ab^	392^a^	46	0.009	0.188	0.250	ns
Shannon	3.02	4.03^c^	3.64^abc^	3.68^bc^	3.64^bc^	3.87^bc^	3.18^ab^	3.78^bc^	2.85^a^	0.21	0.012	0.331	0.081	ns
Chao-1	1,202	1232^abc^	1334^bc^	1221^abc^	1019^abc^	1469^c^	925^ab^	1211^abc^	886^a^	139	0.020	0.158	0.377	ns

* all indices were similar between days 0 and 35 (*p* > 0.05), but both were significantly different (*p* < 0.05) from gut bacteria at day 116 (Table 2). **p-values from lme model with lipid, n-3 and oxygen as fixed effects, interactions between the three and fish tank as a random effect. Differing lowercase letters indicate significant difference (p<0.05).

On day 116, Good’s coverage, number of OTUs, Shannon diversity, and Chao-1 Richness were all significantly affected by n-3 level in the diet while no effects of lipid or oxygen were found (Table 2). In general, alpha-diversity increased when fish were fed low n-3 diets. At high oxygen, fish fed the high lipid diet with low n-3 had the highest diversity and fish fed the high lipid diet with high n-3 had the lowest diversity, which was supported by a significant lipid × n-3 interaction for Shannon diversity. Regarding the environmental samples, Shannon and Chao-1 were different between the gut and tank biofilm (*p* < 0.001) and between the diet and tank biofilm (*p* = 0.001), although no differences existed between the gut and diet (*p* > 0.05). Number of OTUs was more than 10-fold higher in the tank biofilm compared to gut and diet samples.

**Table 2:** Alpha-diversity indices of diet, tank biofilm (*N* = 3), and intestinal bacteria from Atlantic salmon (*N* = 72) fed HL or LL with or without additional n-3 PUFA (+) for 116 days and reared at low and high oxygen.

			Low oxygen				High oxygen				Pooled		*p*-value**		
	Diet	Tank	HL	LL	HL+	LL+	HL	LL	HL+	LL+	SE	Lipid	n-3	Oxygen	X
Coverage (%)	96.3	66.1	98.0^bc^	98.1^bc^	98.2^bc^	98.5^c^	96.6^a^	97.2^ab^	98.6^c^	98.2^bc^	0.4	0.645	<0.001	0.068	n-3 x O
No. of OTUs	584	3,599	370^b^	330^b^	324^b^	284^a^	562^c^	412^bc^	234^a^	349^b^	52	0.960	0.001	0.250	ns
Shannon	3.62	7.60	2.86^bc^	2.47^b^	2.38^ab^	2.64^b^	3.63^c^	2.43^b^	1.67^a^	2.94^bc^	0.24	0.765	0.040	0.857	L x n-3
Chao-1	994	11,548	594^ab^	540^ab^	530^ab^	445^a^	961^c^	795^bc^	425^a^	532^a^	135	0.818	<0.001	0.061	ns

L, lipid; O, oxygen. **p-values from lme model with lipid, n-3 and oxygen as fixed effects, interactions between the three and fish tank as a random effect. Differing lowercase letters indicate significant difference (p<0.05).

### Beta-Diversity of 16S rRNA Gene Sequences

**Figure 3:**
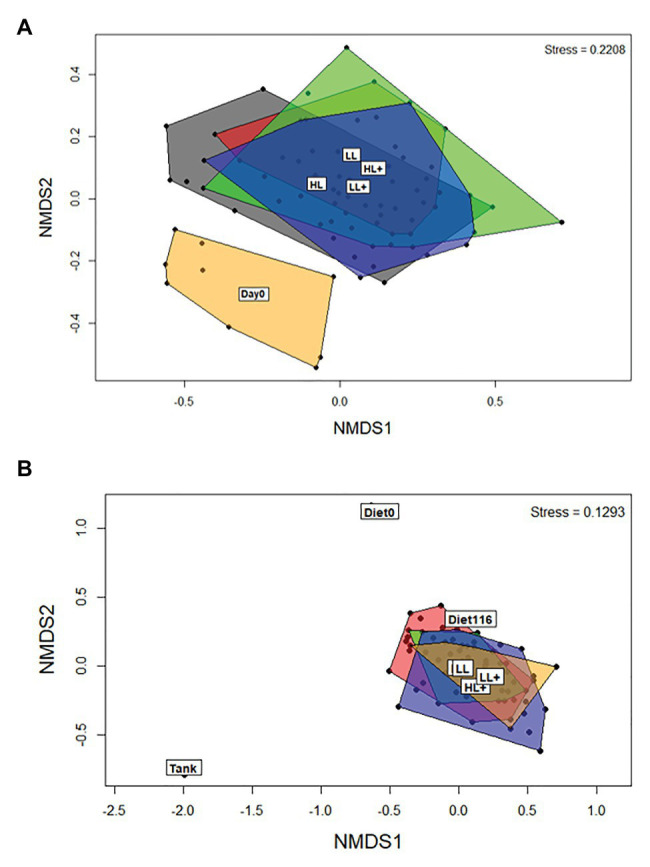
Non-metric dimensional scaling (NMDS) plots of bacterial beta-diversity from the distal intestine of Atlantic salmon fed HL or LL diets with or without additional n-3 PUFA (+). Oxygen conditions were pooled together and fish were sampled on **(A)** day 0 (*N* = 9) and 35 (*N* = 72), and **(B)** day 116 (*N* = 72). Intestine samples were compared to the commercial diet (Diet0), pooled experimental diets (Diet116), and tank biofilm (Tank; *N* = 3).

Between day 0 and 35 there was an effect of day (time) on beta-diversity of gut bacteria (*p* = 0.001), as shown clearly by the separation in the NMDS plot (Figure 3A). On day 35, levels of lipid (*p* = 0.001) and oxygen (*p* = 0.005) affected beta-diversity of gut bacteria, but no effect of n-3 was found (*p* = 0.115) based on ANOSIM (*df* = 68). On day 116, differences in sample type (i.e., gut, diet, and tank biofilm) existed (*p* = 0.001) as clearly shown in the NMDS plot (Figure 3B). Levels of lipid (*p* = 0.009), n-3 (*p* = 0.036), and a lipid × n-3 interaction (*p* = 0.001) affected beta-diversity of gut bacteria, but no effect of oxygen was found (*p* = 0.367) based on ANOSIM (*df* = 71).

**Figure 4:**
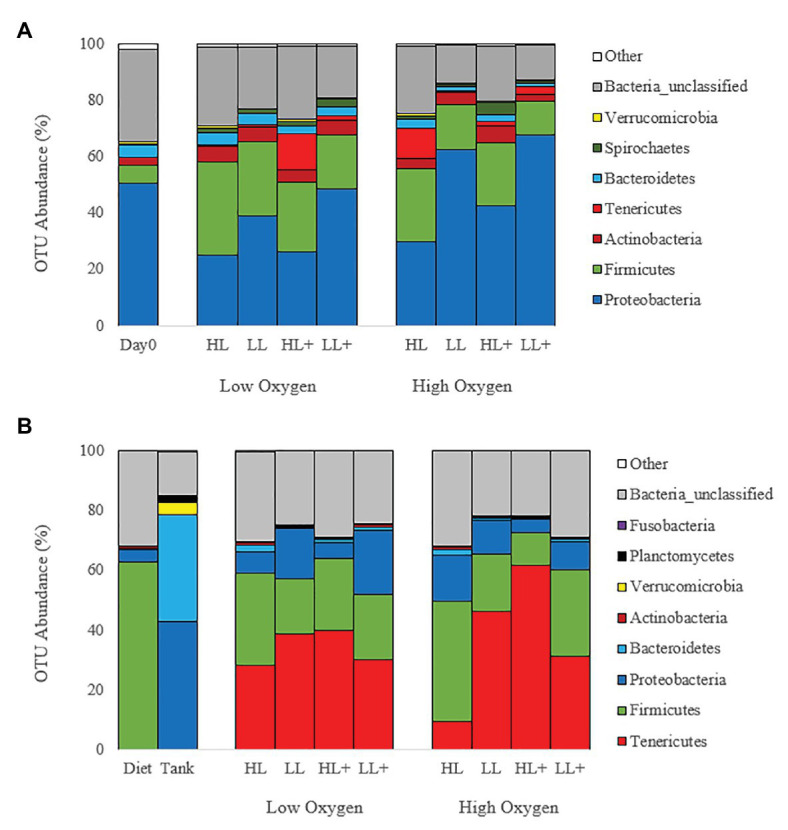
Bar plots of bacteria (phyla level) from the distal intestine of Atlantic salmon fed HL or LL diets with or without additional n-3 PUFA (+) under high or low dissolved oxygen conditions sampled on **(A)** day 0 (*N* = 9), 35 (*N* = 72) and **(B)** day 116 (*N* = 72) along with diet and tank biofilm (*N* = 3).

Mean relative abundance at the phylum level on day 0 and 35 (Figure 4A) were dominated by Proteobacteria (44%) followed by Firmicutes (21%), Actinobacteria (4%), and Tenericutes (3%). On day 116, gut bacteria were dominated by Tenericutes (36%) followed by Firmicutes (24%), Proteobacteria (11%), and Actinobacteria (1%; Figure 4B). Majority of bacteria in the diet were Firmicutes while the tank biofilm consisted of Proteobacteria and Bacteroidetes.

**Figure 5:**
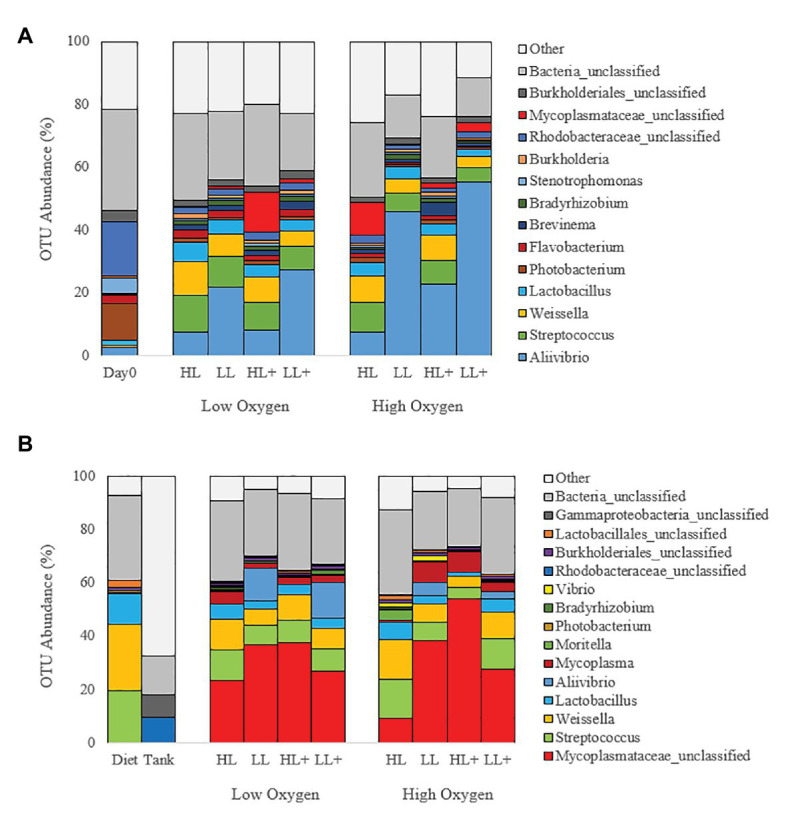
Bar plots of bacteria (genus level) from the distal intestine of Atlantic salmon fed HL or LL diets with or without additional n-3 PUFA (+) under high or low dissolved oxygen conditions sampled on **(A)** day 0 (*N* = 9), 35 (*N* = 72) and **(B)** day 116 (*N* = 72) along with diet and tank biofilm (*N* = 3).

At the genus level, gut bacteria were dominated by *Aliivibrio* (22%) followed by *Streptococcus* (7%), *Weissella* (6%), Rhodobacteraceae (4%), *Lactobacillus* (4%), and Mycoplasmataceae (3%) on day 0 and 35 (Figure 5A). On day 116, gut bacteria were dominated by Mycoplasmataceae (32%), *Streptococcus* (9%), *Weissella* (9%), *Lactobacillus* (4%), *Aliivibrio* (4%), *Mycoplasma* (4%), and Rhodobacteraceae (1%; Figure 5B). Most bacteria in the diet were *Weissella* and *Streptococcus* while the tank biofilm consisted of Rhodobacteraceae and Flavobacteriaceae.

Lactic acid bacteria from the Firmicutes phylum, particularly *Streptococcus* and *Weissella*, were found in higher abundance in the gut of fish fed low lipid and low n-3 diets as analyzed by LEfSe (Table 3). In contrast, fish fed high lipid diets had higher abundance of bacteria in three other phyla: Actinobacteria, Proteobacteria, and Verrucomicrobia.

**Table 3:** Indicator operational taxonomic units (OTUs) of intestinal bacteria from Atlantic salmon (*N* = 72) on day 116 that explain differences between dietary treatments identified by Linear discriminant analysis effect size (LEfSe).

Phyla	Order	Genus	Diet	LDA	*p*-value
Actinobacteria	Actinomycetales	Microbacteriaceae_unclassified	High Lipid	2.0	0.033
Firmicutes	Lactobacillales	Lactobacillales_unclassified	Low Lipid & Low n-3	2.1/2.3	0.043/0.003
		*Lactobacillus*	Low n-3	2.3	0.035
		*Leuconostoc*	Low n-3	3.0	0.033
		*Streptococcus*	Low Lipid & Low n-3	2.3/3.8	<0.001/0.041
		*Weissella*	Low Lipid & Low n-3	2.7/2.9	0.034/0.012
Proteobacteria	Burkholderiales	Burkholderiales_unclassified	Low Lipid & Low n-3	2.4/2.1	<0.001/0.033
	Enterobacteriales	Enterobacteriaceae_unclassified	High Lipid	2.4	0.025
	Oceanospirillales	Chromohalobacter	High Lipid	2.9	0.048
	Vibrionales	*Aliivibrio*	Low Lipid	4.5	<0.001
		*Photobacterium*	Low n-3	2.8	0.021
Verrucomicrobia	Verrucomicrobiales	Verrucomicrobiaceae_unclassified	High Lipid	2.6	0.030

LDA; linear discriminant analysis.

### Functional Predictions of 16S rRNA Metagenome

**Figure 6:**
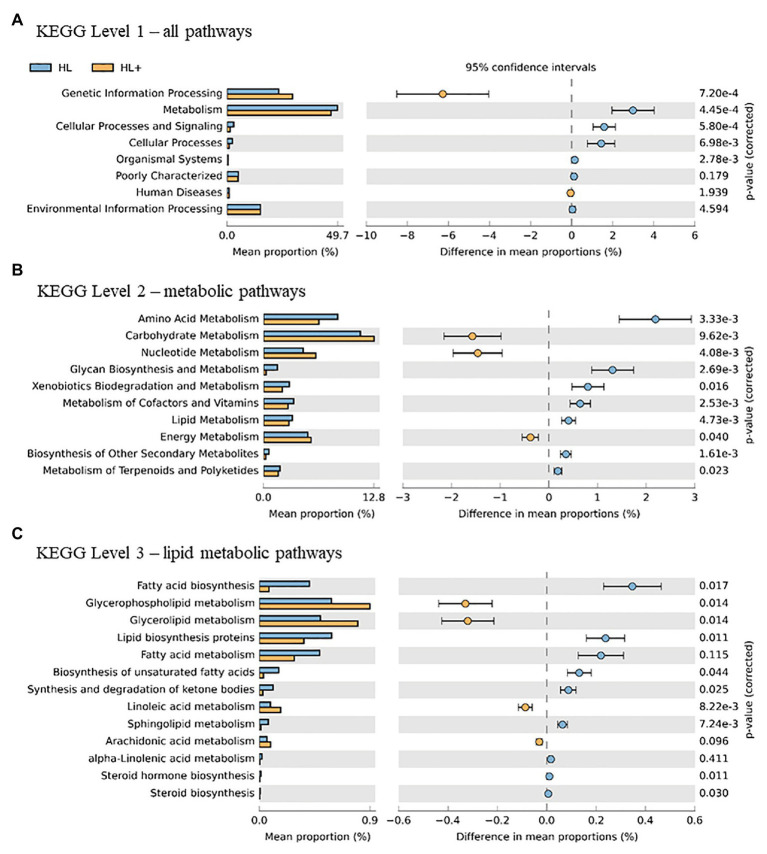
Statistical Analysis of Taxonomic and Functional Profiles (STAMP) plots of predictive metagenomic analysis using phylogenetic investigation of communities by reconstruction of unobserved states (PICRUSt) to infer function of the distal intestine microbiome (*N* = 72) of Atlantic salmon on day 116 fed HL and high lipid with additional n-3 LC-PUFA (HL+). Kyoto Encyclopedia of Genes and Genomes (KEGG) Level 1 includes all pathways **(A)**, two includes metabolic pathways **(B)**, and three includes lipid metabolic pathways **(C)**. *p-*values were generated by Welch’s *t*-test with Bonferroni correction and <0.05 was considered significant.

**Figure 7:**
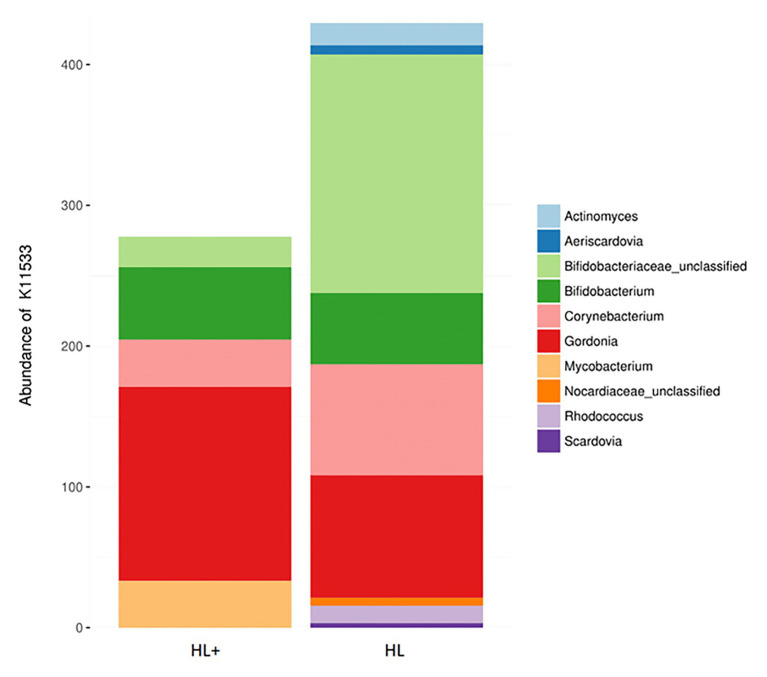
Contribution of bacterial OTUs to the abundance of KEGG ortholog K11533 (fatty acid synthase – see Figure 6C) in the gut of Atlantic salmon (*N* = 72) fed high lipid with additional n-3 LC-PUFA (HL+) or HL.

Overall, the predictive metagenomic pathways at KEGG Level 1 from the gut bacteria were dominated by metabolism and genetic information processing and to a lesser extent by environmental information processing and cellular processes (Figure 6A). Gut bacteria in fish fed high lipid diet with high n-3 showed increased (*p* < 0.001) functional pathways of genetic info processing while high lipid diet with low n-3 showed activation of metabolic and cellular processing pathways. Within the metabolic pathway at KEGG Level 2, the metabolism of carbohydrates, amino acids, and nucleotides dominated followed to a lesser extent by energy, lipids, and vitamins (Figure 6B). The high lipid diet with high n-3 elevated (*p* < 0.001) metabolic pathways related to carbohydrates and nucleotides, whereas the high lipid diet with low n-3 elevated amino acid, vitamin, and lipid metabolic pathways. Within the lipid metabolic pathway at KEGG Level 3, the high lipid diet with n-3 elevated (*p* < 0.05) metabolic pathways of glycerolipids, phospholipids, linoleic acid (18:2n-6), and arachidonic acid (20:4n-6; *p* = 0.096), whereas the high lipid (HL) diet elevated (*p* < 0.05) the biosynthesis of fatty acids, unsaturated fatty acids, lipid proteins, and steroids (Figure 6C). Alpha-linolenic acid (18:3n-3) was elevated in fish fed the HL diet, but was not significant (*p* = 0.411). A higher abundance of Bifidobactericeae, *Actinomyces*, and *Rhodococcus* resulted in higher pathway activation of fatty acid synthase (Figure 7). There were no significant effects between high and low lipid or oxygen treatments (data not shown).

## Discussion

There has been a spike in the number of studies using high-throughput sequencing to characterize the gut microbes of salmonids including farmed species of Atlantic salmon (Zarkasi et al., 2014, 2016; Gajardo et al., 2016, 2017; Dehler et al., 2017; Rudi et al., 2018; Villasante et al., 2019), rainbow trout (Huyben et al., 2017, 2018, 2019; Lyons et al., 2017a,b,c; Michl et al., 2017; Bruce et al., 2018) and Arctic charr (Nyman et al., 2017). Of these, most dietary studies have investigated replacing fishmeal with vegetable, insect, or microbial protein sources. Similar to the present study, a study by Zarkasi et al. (2016) fed diets with high (300:400 g/kg) and low (200:500 g/kg) ratios of lipid:protein to Atlantic salmon post-smolts for 5-months and found that alpha-diversity of gut microbiota increased for fish fed high lipid diets, which decreased across all treatments over time. Declining alpha-diversity has been previously correlated with age as fish tend to have a reduced ability to filter microbial communities as they mature (Llewellyn et al., 2016; Burns et al., 2018; Heys et al., 2020). Two studies have examined the effect of replacing fish oil with vegetable oil on the gut microbiota of salmonids that drastically change the intake level of n-3 LC-PUFA, e.g., EPA and DHA (Ingerslev et al., 2014; Rudi et al., 2018). Compared to our results, Rudi et al. (2018) found similar alpha-diversity and beta-diversity in the gut of Atlantic salmon smolts, especially the high abundance of *Streptococcus*, *Weissella*, *Lactobacillus*, *Photobacterium*, and *Bradyrhizobium*. However, the present study is unique in that it is the first to investigate the effect of both lipid:protein and levels of n-3 LC-PUFA as well as hypoxia on the microbiota of a salmonid species.

The effect of dietary n-3 PUFA on alpha-diversity (i.e., Shannon and Chao-1 indices) of gut microbes on day 116 (Table 2) has not been observed before in salmonid fishes. This was partially in agreement with our original hypothesis that alpha-diversity would decrease with higher n-3 LC-PUFA levels, which was only the case at the end of the trial. In humans, 45 year-old men that consumed 600 mg of n-3 PUFA every day over 2 weeks resulted in a reduced alpha-diversity of their gut microbes (Noriega et al., 2016). These authors attributed the decrease to a higher abundance of bacteria that produce short-chain fatty acids, such as *Lactobacillus*, although in the present study, we found a reduction of this genus and lower activation of fatty acid biosynthesis pathways in gut bacteria when fed high n-3 LC-PUFA diets (Figures 5, 6C). In gilthead seabream, feeding a low protein diet with high n-3 for 81 days had the lowest Shannon diversity of gut microbes sequenced from extracted bands from PCR-DGGE (Castro et al., 2019). In Atlantic salmon, Rudi et al. (2018) found that replacing fish oil with vegetable oil, low in n-3, had no effect on Shannon diversity of gut microbes in both freshwater and saltwater. In rainbow trout, Ingerslev et al. (2014) reported that replacing fish oil with rapeseed oil had no effect on Shannon diversity of gut microbes. However, both salmonid studies fed fish vegetable oil diets for only 20 and 23 days and the present study also confirmed that similarly there was no effect of n-3 on alpha-diversity on day 35 (Table 1). In contrast, lipid level only influenced alpha-diversity in the short-term and was not evident at the end of the trial on day 116 (Table 2 and Figure 2). This agrees with a 5-month study by Zarkasi et al. (2016) that found alpha-diversity of gut bacteria was higher in Atlantic salmon fed a high lipid (low protein) diet compared to a control diet, although after some months, the diversities were equal (Zarkasi et al., 2016). One theory may be that alpha-diversity decreases over time and masks effect of large macronutrients, such as lipid, whereas n-3 influences metabolic pathways that require more time to alter gut microbial composition. On day 116 in the present study, Shannon diversity of gut microbiota were the highest in fish fed high lipid diets whereas diets with high n-3 suppressed diversity and indicated a lipid × n-3 interaction (Table 2). In a parallel study, Huyben et al. (2021) found that the digestibility of n-3 LC-PUFA was 99–100% compared to 95–98% of lipid for Atlantic salmon, which suggests that the amount of leftover lipid in the feces would influence the gut microbiota more than leftover n-3 and may have resulted in a delayed effect. Results from the present study indicate that alpha-diversity of gut microbes can be influenced in the short-term (<35 days) by feeding different lipid levels while changes in n-3 levels only became evident after long-term feeding (36–116 days) and may have suppressed the beneficial effects of high lipid levels.

Changes in the beta-diversity (i.e., relative abundance) of gut bacteria due to changes in dietary n-3 PUFA level have been previously reported. Ingerslev et al. (2014) found that replacing fish oil with vegetable oil increased Firmicutes:Proteobacteria ratio, specifically genera of *Streptococcus*, *Leuconostoc*, and *Weissella*, which were also found in the present study (Table 3; Figures 4, 5). However, the diet also included high amounts of pea protein concentrated to replace fishmeal, thus the effects of the rapeseed oil on the gut microbiota are uncertain. In the present study, only a small volume of fish oil was replaced with olive oil in order to test the change in n-3 LC-PUFA rather than the oil source. Similar to our results, Zarkasi et al. (2017) found that *Aliivibrio*, *Vibrio*, and *Photobacterium* were consistently dominant genera despite feeding different diets containing high and low lipid levels (200 and 300 g/kg) to an *in vitro* salmon gut system. *In vivo*, Zarkasi et al. (2016) found that the high lipid:protein diet reduced the abundance of Proteobacteria, specifically Gammaproteobacteria (class level) and *Aliivibrio*, in the gut of Atlantic salmon that was similar to the present study (Table 3). In the present study, genera of *Streptococcus*, *Leuconostoc*, and *Weissella* were the most abundant in the diet and had similar abundance in the gut at day 35 and 116, which indicates a direct diet-gut connection in terms of bacteria colonization (Figure 5). In contrast, OTUs of Rhodobacteraceae and Flavobacteriaceae from the tank biofilm were not present in the gut at a high abundance (<0.1%).

Lactic acid bacteria have been identified as beneficial gut microbes; therefore, higher abundance in fish fed low lipid and low n-3 diets may indicate a more favorable bacterial community (Table 3; Figure 5). The genera of *Streptococcus*, *Leuconostoc*, and *Weissella* are lactic acid bacteria (Lactobacillales order) that are generally considered as favorable gut microbes due to their abilities to stimulate and enhance gut development, digestive function, mucosal tolerance, immune response, and disease resistance (Merrifield et al., 2010; Ringø et al., 2018). Our results agree with an earlier study that showed feeding a marine diet with high PUFA (i.e., linoleic acid) depressed the viable counts of lactic acid bacteria, specifically *Lactobacillus*, in the gut of Arctic charr (Ringø, 1993). The intact lipopolysaccharide layer of Gram-negative microbes apparently screens the cells against medium and long-chain fatty acids and prevents their accumulation on the inner cell membrane, whereas Gram-positive microbes (e.g., lactic acid bacteria) cannot and become inhibited (Sheu and Freese, 1973). Suppression of lactic acid bacteria by high inclusions of n-3 LC-PUFA in the diet may reduce gut health; therefore, gut microbiota needs to be considered when optimizing n-3 levels for optimal growth performance of Atlantic salmon.

The increase in abundance of Tenericutes, specifically *Mycoplasma* (genus) and Mycoplasmataceae (order), in the fish gut from day 35 to 116 has been shown previously. Zarkasi et al. (2014) found higher abundance of *Mycoplasma*, *Photobacterium*, *Aliivibrio*, and *Vibrio* after 13 months of rearing Atlantic salmon in Tasmanian sea-cages and increasing dietary lipid from approximately 230–330 g/kg. In contrast, abundance of *Aliivibrio* was lower at the end of the trial and lower in fish fed high lipid diets in the present study (Table 3; Figure 5B). Other studies have found Tenericutes or *Mycoplasma* as a common inhabitant in the gut at high abundances of up to 83% in rainbow trout (Lyons et al., 2017c; Huyben et al., 2018) and 92% in Atlantic salmon (Zarkasi et al., 2014; Llewellyn et al., 2016; Rudi et al., 2018). The most common species of *Mycoplasma* found in the present study was *Mycoplasma penetrans*, which has been highlighted as a candidate agent of transmissible tumors in the gut of zebrafish (Burns et al., 2018). These authors found that intestinal neoplasm was transmitted from afflicted to healthy fish through cohabitation while acquiring an increased abundance of *Mycoplasma*, indicating an infectious etiology rather than tumors caused by diet, water quality, or genetic background. Heys et al. (2020) found that *Mycoplasma* are not transient in the gut of Atlantic salmon but rather adapt to their host environment. In addition, both tumors and *Mycoplasma* have been found in the gut of older zebrafish and wild Atlantic salmon (Paquette et al., 2013; Llewellyn et al., 2016), as was the case in the present study as *Mycoplasma* was not abundant until day 116 (Figure 5). Despite the potential pathogenic nature of *Mycoplasma*, it can persist in healthy fish populations without clinical signs of disease (Burns et al., 2018), which was reflected in the present study since fish fed high lipid diet with high n-3 had the highest final weight and abundance of *Mycoplasma* and Mycoplasmataceae (Figure 5B). These fish also had reduced alpha-diversity in the gut, which has been associated with higher incidence of disease (Li et al., 2016; Parshukov et al., 2019). One theory may be that fish do not rely on the nutrient production from gut microbes as much if requirements are met by the diet consumed. In fish fed the high lipid diet with high n-3, gut microbes had a higher activation of energy and carbohydrate metabolic pathways (Figure 6) that may represent a shift from specific nutrient production to energy acquisition. For instance, fish fed high lipid diets with low n-3 had higher metabolism of amino acids, vitamins, and lipids possibly in lieu of energy metabolism. More research is needed to determine the impacts of *Mycoplasma*, alpha-diversity, and predictive metagenomic function of gut microbes on the health and growth performance of fish.

Previous studies have found that the metabolism contributes the most to the predictive pathways of the salmonid gut microbiome, followed by information and cellular processing (Dehler et al., 2017; Lyons et al., 2017c; Yildirimer and Brown, 2018). Specifically, our results agree that a high proportion of pathways are attributed to amino acid, carbohydrate, and energy metabolism and to a lesser extent vitamin and lipid metabolism (Dehler et al., 2017; Lyons et al., 2017c). Very few studies exist that compare the impact of the gut microbiome on lipid metabolism in fish, despite a connection between n-3/n-6 fatty acids and gut microbes. A study by Yildirimer and Brown (2018) found that a population of wild rainbow trout had lower lipid absorption and higher activation of lipid metabolism pathways, specifically linoleic acid (18:2n-6). Wild fine flounder (*Paralichthys adspersus*) were found to have higher activation of fatty acid metabolism and biosynthesis of unsaturated fatty acids pathways compared to farmed flounder presumably fed a formulated diet higher in lipid and LC-PUFA (Ramírez and Romero, 2017). These results agree with the current study where fish fed the high lipid diet with low n-3 LC-PUFA resulted in higher activation of lipid metabolism, specifically the biosynthesis of saturated and unsaturated fatty acids (Figures 6B,C). Other than glycerolipid metabolism, the only lipid metabolic pathway significantly affected by the high n-3 diet was the metabolism of n-6 PUFAs linoleic acid (18:2n-6) and arachidonic acid (20:4n-6), which may be explained by higher levels in the diet. The pathway for fatty acid synthase was dominated by Actinobacteria, such as *Bifidobacterium*, *Corynebacterium*, and *Gordonia* (Figure 7), which have <1% abundance in the gut but may be key contributors to lipid metabolism. In Arctic charr, feeding marine oil reduced viable counts of *Corynebacterium* compared to fish fed soybean and linseed oils (Ringø et al., 2002). In humans, consuming n-3 PUFA supplements increased abundance of *Bifidobacterium* (Watson et al., 2018), which produces short-chain fatty acids (i.e., butyrate) that can act as an energy source, improve cell signaling, and reduce inflammation in the gut (Noriega et al., 2016). These findings suggest that the gut microbiome can respond in efforts to compensate for a lack of n-3 in the diet by increasing the abundance of fatty-acid producing bacteria. However, these results are only functional predictions based on existing reference genomes and deep shotgun sequencing of all genomes in each sample is the preferred technique (Langille et al., 2013).

We did not expect that a reduction in dissolved oxygen from 8.0 to 6.7 mg/L (93–78% saturation) would have minor or no effects on viable plate counts, alpha-diversity, beta-diversity, indicator OTUs (LEfsE), or predictive metagenomics function of gut bacteria in Atlantic salmon. There was an initial effect of oxygen on viable plate counts and beta-diversity that may be due to reduced feed intake at the start of the experiment, while no effect of oxygen was found on day 116. In contrast, reduced oxygen from 6–7 to 3–4.5 mg/L resulted in lower alpha-diversity, different beta-diversity, altered metabolism pathways (e.g., fatty acid), and indicator OTUs of Chromatiales, Enterobacteriales, and Spirochaetales in the gut of four-eyed sleeper (*Bostrychus sinensis*; Fan et al., 2020). Reduced oxygen from 6.5 to 2.5 mg/l resulted in lower alpha-diversity, different beta-diversity, altered metabolism pathways (e.g., linoleic acid), and indicator OTUs of *Clostridia* and *Lactobacillus* in the gut of oriental river prawn (*Macrobrachium nipponense*; Sun et al., 2020). Reduced oxygen from 9.7 to 8.6 mg/L resulted in lower alpha-diversity and abundance of *Lactobacillus* in the gut of rainbow trout, although temperature was also a confounding factor in that study as it increased from 11 to 18°C (Huyben et al., 2018). Therefore, in the present study, the reduction in dissolved oxygen we believe was not severe enough to cause significant changes in gut microbiota of Atlantic salmon.

## Conclusion

This study investigated the effects of two dietary components, lipid, and n-3 LC-PUFA level, as well as a common chronic stressor, hypoxia, on the gut microbiome of Atlantic salmon using agar plating, 16S rRNA gene next-generation sequencing, and bioinformatic and predictive metagenomic analyses. This is the first study to find significant effects of both dietary n-3 and a lipid × n-3 interaction on the alpha and beta-diversities of gut bacteria in salmonids. There were stark differences in the effects of dietary lipid and n-3 levels on gut microbes between days 0, 35, and 116. Initially, high lipid diets (low protein) increased alpha-diversity and by the end of the trial high n-3 levels reduced alpha-diversity, possibly suppressing beneficial effects of high lipid. There was a general reduction of alpha-diversity over time. Between diets, beta-diversity and phyla abundance were similar, except for some genera, such as lactic acid bacteria (e.g., *Streptococcus*, *Weissella*, and *Lactobacillus*), associated with fish fed low lipid (high protein) and low n-3 diets. Between time points, the relative abundance of the Tenericutes phylum (i.e., *Mycoplasma*) increased 10-fold to approximately 36% of gut microbes, which supports previous findings that older fish are less able to filter pathogenic gut bacteria. At high oxygen, fish fed the high lipid diet with high n-3 (closest to a commercial analogous diet) had reduced alpha-diversity, lowest abundance of lactic acid bacteria (11%), and highest abundance of *Mycoplasma*/Mycoplasmataceae (62%). The only effect of oxygen was on the viable counts of bacteria and an initial effect on beta-diversity; otherwise, the reduction in dissolved oxygen from 8.0 to 6.7 mg/l may have been too minor to influence the alpha and beta-diversities or predictive functions of gut microbes at the end of the trial. These findings suggest these fish may have a poor gut microbiota or dysbiosis even though this group had the largest final fish weight, thus more research needs to determine the function of *Mycoplasma* and relation to fish growth performance. With regard to predictive function, PICRUSt analysis showed that feeding lower levels of n-3 resulted in higher metabolism of saturated and unsaturated fatty acids by the gut microbiome, potentially as an effort to compensate for low n-3 in the diet. In summary, our results indicate that the viable plate counts, alpha-diversity, beta-diversity, and predictive function of gut bacteria in Atlantic salmon post-smolts are influenced by dietary lipid:protein ratio and n-3 LC-PUFA levels over time, with only minor effects of dissolved oxygen.

## Data Availability Statement

The original contributions presented in the study are publicly available. This data can be found here: https://www.ncbi.nlm.nih.gov/bioproject/PRJNA650141.

## Ethics Statement

The animal study was reviewed and approved by Animal Welfare and Ethical Review Body (reference AWERB/1617/84), UK Home Office.

## Author Contributions

The experiment was planned by BG, DH, and BRu. The fish were measured and sampled for intestinal content by DH, BG, and BRo. The lab work and sequencing on the 16S rRNA diversity was done by DH and BRo. Bioinformatics, statistics, and metagenomic analysis were done by DH and MB. The manuscript was written by DH and all co-authors contributed revisions and approved the final version.

### Conflict of Interest

The authors declare that the research was conducted in the absence of any commercial or financial relationships that could be construed as a potential conflict of interest.

## Footnotes

^1^https://www.ncbi.nlm.nih.gov/bioproject/PRJNA650141
